# Heterogeneity and versatility of the extracellular matrix during the transition from pleomorphic adenoma to carcinoma ex pleomorphic adenoma: cumulative findings from basic research and new insights

**DOI:** 10.3389/froh.2023.942604

**Published:** 2023-04-17

**Authors:** João Figueira Scarini, Reydson Alcides de Lima-Souza, Luccas Lavareze, Maria Clara Falcão Ribeiro de Assis, Ingrid Iara Damas, Albina Altemani, Erika Said Abu Egal, Jean Nunes dos Santos, Ibrahim Olajide Bello, Fernanda Viviane Mariano

**Affiliations:** ^1^ Department of Pathology, Faculty of Medical Sciences, University of Campinas (UNICAMP), Campinas, Brazil; ^2^ Department of Oral Diagnosis, School of Dentistry, University of Campinas (FOP/UNICAMP), Piracicaba, Brazil; ^3^ Biorepository and Molecular Pathology, Huntsman Cancer Institute, University of Utah (UU), Salt Lake City, UT, United States; ^4^ Department of Oral and Maxillofacial Pathology, School of Dentistry, Federal University of Bahia, Salvador, Brazil; ^5^ Department of Oral Medicine and Diagnostic Sciences, College of Dentistry, King Saud University, Riyadh, Saudi Arabia

**Keywords:** extracellular matrix (ECM), pleomorphic adenoma (PA), carcinogenesis, carcinoma ex pleomorphic adenoma (CXPA), review

## Abstract

Pleomorphic adenoma (PA) is the most common salivary gland tumor, accounting for 50%–60% of these neoplasms. If untreated, 6.2% of PA may undergo malignant transformation to carcinoma ex-pleomorphic adenoma (CXPA). CXPA is a rare and aggressive malignant tumor, whose prevalence represents approximately 3%–6% of all salivary gland tumors. Although the pathogenesis of the PA-CXPA transition remains unclear, CXPA development requires the participation of cellular components and the tumor microenvironment for its progression. The extracellular matrix (ECM) comprises a heterogeneous and versatile network of macromolecules synthesized and secreted by embryonic cells. In the PA-CXPA sequence, ECM is formed by a variety of components including collagen, elastin, fibronectin, laminins, glycosaminoglycans, proteoglycans, and other glycoproteins, mainly secreted by epithelial cells, myoepithelial cells, cancer-associated fibroblasts, immune cells, and endothelial cells. Like in other tumors including breast cancer, ECM changes play an important role in the PA-CXPA sequence. This review summarizes what is currently known about the role of ECM during CXPA development.

## Introduction

Pleomorphic adenoma (PA) is the most common salivary gland tumor (SGT), accounting for 50%–60% of these neoplasms (1). If untreated, it is estimated that up to 6.2% of PAs undergo malignant transformation to carcinoma ex pleomorphic adenoma (CXPA) (2). Multiple recurrences, male sex, advanced age, previous radiation therapy, and tumor size are the most frequently reported risk factors for the malignancy process (1, 3, 4). CXPA can be characterized as a rare and aggressive malignant tumor (5), that represents approximately 3%–6% of all SGTs and presents a 5-year survival of around 63% (6, 7). Most of them affect the parotid glands, usually manifesting in adult women between 50 and 70 years old (5, 7). Recent studies showed that genomic instability (8, 9), metabolic shifts (10), and changes in the tumor microenvironment (TME), mainly governed by the myoepithelial cell (11), are the most important molecular events oin the CXPA carcinogenesis.

**Figure 1:**
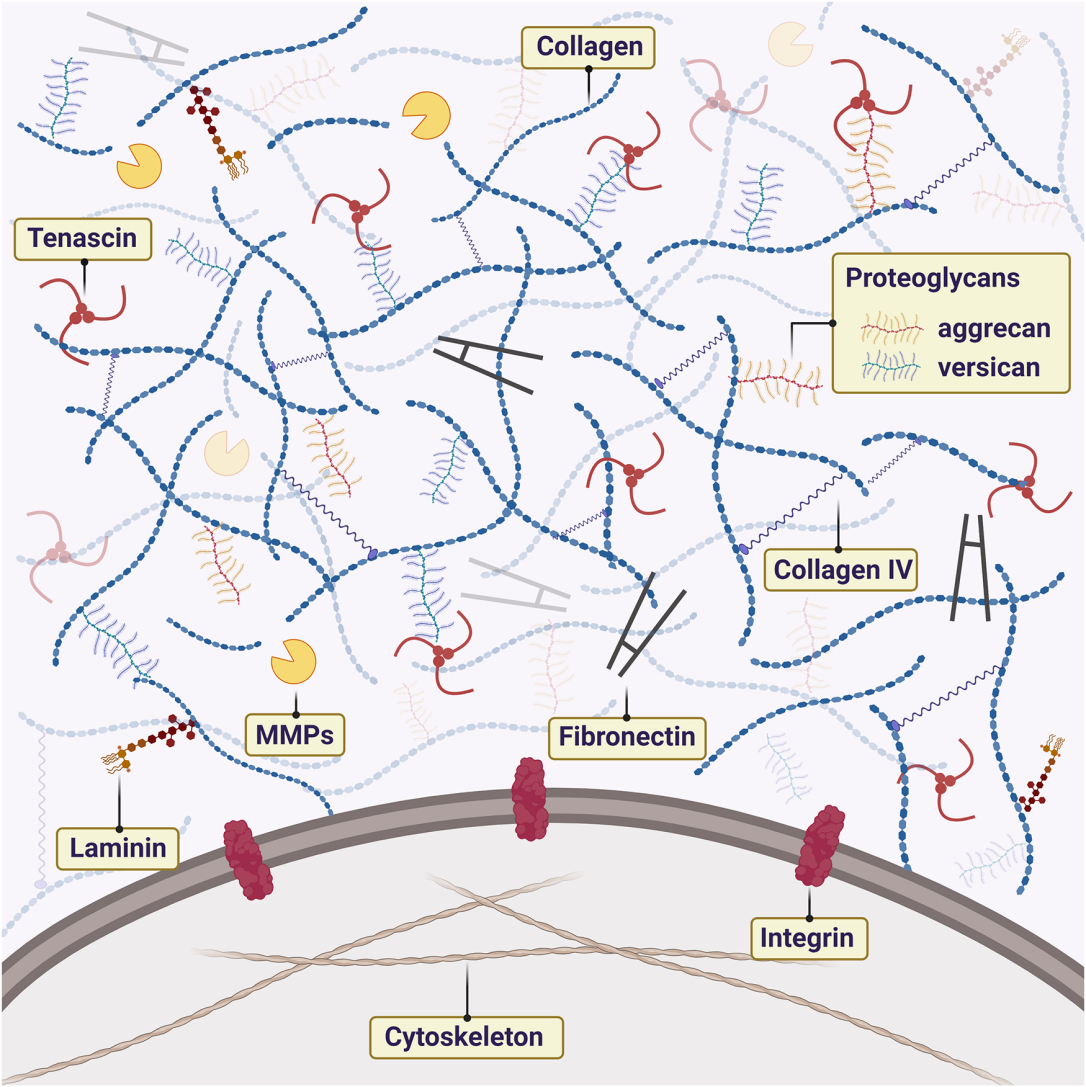
Schematic illustration of extracellular matrix (ECM) components in the tumor microenvironment of PA and CXPA. ECM is rich in macromolecules such as laminins, proteoglycan (PG) complex, collagen, and fibronectin. The interaction between cells and ECM is mainly mediated by cell receptors of the matrix components, such as integrins. Metalloproteinases (MMPs) promote the degradation and remodeling of ECM, favoring cell proliferation.

TME is an active component that is in constant remodeling (12–14). Neoplastic cells can regulate the microenvironment to promote cell survival (15, 16). The role of the TME is now becoming appreciated as an important component in cancer development, which is driven by interactions between tumor cells and their microenvironment. Many reports have stated that is possible to reprogram genotypically malignant cells into phenotypically normal cells by manipulating the TME and inhibiting signaling pathways (17, 18). The extracellular matrix (ECM) is the non-cellular component of the TME composed of assembled macromolecules such as laminins, proteoglycan (PG) complex, collagen, integrins, and cadherins. Several studies have focused on the ECM etiology and genesis of neoplasms to provide a targeted therapeutic basis (16, 17, 19–21). The highly organized structure of the ECM is also home to important biomolecules and growth factors, as well as biomechanical forces that can modulate cancer cells to promote the metastatic cascade (22). PA and CXPA are tumors that present a dense and abundant ECM with high amounts of PGs and collagens (Figure 1). In the PA-CXPA sequence, ECM is especially interesting as its structure and components change throughout the PA malignant transformation, showing a potential promoting role in the CXPA progression and invasion.

In this review, we provided an overview of the key components of the ECM and their degradation enzymes aiming at improving our knowledge of the mechanisms contributing to the progression of the PA-CXPA sequence and CXPA dissemination. We also discussed ECM-based diagnosis as well as the potential new approaches for direct and complementary therapeutic targets for this tumor.

## ECM in PA-CXPA progression

### Proteoglycans

Proteoglycans (PGs) are macromolecules formed by a central protein in which one or more chains are covalently linked (23). PGs are important in modulating the mechanical properties of ECM, including its rigidity (24). The remodeling of the ECM represents an important factor in tumor development as significant modification in the synthesis of PGs occurs (25–27). ECMs enriched with PGs are associated with malignant transformation, inflammatory infiltration in the TME, and tumor aggressiveness (24, 28). PGs are implicated in cell migration/invasion and alteration of TME in different tumors such as colorectal carcinoma, esophageal carcinoma, hepatocellular carcinoma, and breast carcinoma (29–32). In all these carcinomas, targeting PGs may provide new therapeutic approaches in the future.

Regarding PA, recently a systematic literature review highlighted the importance of PGs in tumor development (33). Their findings showed that while normal salivary gland myoepithelial cells cannot secrete PGs, neoplastic myoepithelial cells of PA produce numerous PGs reflecting tumor development and its biological behavior (33). Xylosyltransferase II (XT-II), an isoform of xylosyltransferase I (XT-I) is involved in the initial step of PGs biosynthesis. Silencing of XT-I and XT-II genes *via* RNAi blocked PGs biosynthesis in neoplastic myoepithelial cells from the primary culture of salivary gland PA, leading to inhibition of invasion, migration, and tumor implantation into the fibroblast framework (34, 35). These findings provide evidence for the crucial role of PGs in the formation of the PA tumor stroma (Table 1). Indeed, over time, several PGs have been identified and correlated with PA ECM including lumican, chondromodulin-I, chondroitin 4S, chondroitin 6S, dermatan sulfate, heparan sulfate, and keratan sulfate (36–40). In this regard, it is worth citing syndecan-1 (CD138), a member of the cell surface heparan sulfate PGs family. CD138 has been reported in several types of tumors, including breast, urinary bladder, gallbladder, pancreatic, ovarian, endometrial, and prostate cancer, and normal tissues (41). In PA, however, CD138 expression did not present any correlation regarding the tumor behavior (42). Further studies, however, are encouraged to understand the role of CD138 in the malignant transformation of PA.

**Table 1:** Potential role of the ECM during the transition from PA to CXPA adenoma.

ECM component	PA	CXPA
Proteoglycans	Luminal and abluminal cells express proteoglycans. Tenascin is most expressed by epithelial cells	Loss of expression in epithelial cells, with strong positivity in myoepithelial cells, conferring the myoepithelial cell potential for tumor promotion
Collagen	Different types of collagens compose the extracellular matrix of the PA, conferring its morphological heterogeneity and advantage in cell proliferation	Myoepithelial cells increase the production of type I collagen, especially on the invasive front. Collagen type IV is correlated with CXPA metastatic behavior
Integrin	Usually expressed in the luminal and abluminal cells. Its role has been little explored in the PA	Although scarcely explored in CXPA, lower expression in more advanced salivary carcinomas is correlated with invasiveness
Laminin	Usually expressed in the ECM, conferring advantages in cell proliferation	Laminin is expressed in benign myoepithelial cells surrounding nests of malignant epithelial cells. It plays an important role in the early stages of the carcinogenic process
Fibronectin	Usually expressed in the ECM. Its role was little explored in the PA	Although it may suppress the tumor suppressor function of myoepithelial cells in carcinomas *in situ*, in the early stages of CXPA, invasive front cells lose fibronectin expression
Cadherin	In PA, it may be correlated with tumor recurrence	Loss of E-cadherin expression in malignant cells is correlated with advantages in cell proliferation and invasion
MMPs and TIMPs	Myoepithelial cells express MMP-2 and MMP-9, which leads to the degradation of the cell membrane and contact of the myoepithelial cells with the tumor stroma	Myoepithelial cells increase the expression of MMP-2 and MMP-9 and decrease the expression of TIMP-2, contributing to tumor invasion

**Legends**: CXPA, Carcinoma ex pleomorphic adenoma; ECM, Extracellular matrix; MMP, Metalloproteinases; PA, Pleomorphic adenoma; TIMP, Tissue inhibitors of metalloproteinases.

Extremely common, the presence of myxoid stroma in PA has already been correlated with recurrence (43). In this regard, among many other structural components, the myxoid stroma of the PA is rich in perlecan (37). This PG acts in the signaling of such growth factors as bFGF (44). In PA, the presence of perlecan in the myxoid stroma was critical for capsular invasion, and vascular involvement of the neoplasm (45). We believe that the study of perlecan in the matrix in the PA-CXPA sequence may provide important evidence for the tumor invasion of CXPA and, consequently, distant metastasis of this neoplasm.

Hyaluronan (HA) and its binding molecules, cartilage binding protein (LP), and PGs (as aggrecan and versican) are structural components of ECM (46, 47). HA-LP-aggrecan complexes are present in the chondromyxoid matrix of the PA, but not in malignant SGTs. In histopathological analysis, this finding is crucial and may assist in distinguishing *de novo* carcinomas from those originating in a PA (48). Indeed, aggrecan has long been reported as an important character of the epithelial-mesenchymal transition (EMT) and tissue heterogeneity in PA (49, 50). While versican is specific for malignant neoplasms (51), in PA it may be present in mesenchymal fibrous areas and some myoepithelial cells (48). This finding may indicate an area susceptible to PA malignant transformation.

Homing cell adhesion molecule (CD44) is a transmembrane PG related to cell-cell adhesion and cell-ECM adhesion. It interacts with several ligands, including hyaluronic acid, collagen, FN, and laminin (52). CD44 has been reported as an important character for PA tumor initiation (53). Curiously, while the CD44 gene is overexpressed in PA (54), the CD44 protein is downregulated (55–57). CD44 protein expression is increased as malignant transformation takes place (58, 59). Different results, however, can be found in the literature. The use of different isoforms and heterogeneity in analysis among different studies may justify the reduced expression of CD44 in CXPA in other studies (56, 60). CD44 has been recognized as one of the markers of multipotency of neoplastic cells in SGTs (61) and this sheds light on its potential as a therapeutic target for the treatment of CXPA.

In several cancers, including breast and colon carcinomas, the tenascin family can be considered an important marker of tumor progression (62). The expression of tenascin in PA is related to tumor expansion (63). TN is observed in the stromal compartment, being more pronounced in the denser stromal and chondroid areas than in the myxoid and hyaline areas (64, 65). The expression of tenascin in chondroid areas could be explained by the fact that tenascin binds to chondroitin sulfate proteoglycans of the matrix (62). Chondromyxoid differentiation in the PA may be induced by tenascin (66). It is important to note that tenascin in the PA is usually present among the epithelial tumor cells that form the ductiform structures and are therefore not in direct communication with the supporting stroma (67). Other groups had already suggested that tenascin is produced by epithelial cells of the SGTs (68).

Interestingly, during PA malignant transformation, this pattern is altered. Tenascin expression is absent in the malignant ductal epithelial cells of the CXPA while a strong and diffuse positivity is present in the cytoplasm of the CXPA malignant myoepithelial cells (69). These findings indicate the importance of tenascin as a contributor to the myoepithelial cell-promoting potential in the CXPA (11). Indeed, a key promoting role of tenascin in the CXPA progression was evidenced in the study by Félix and collaborators (2004) where all cases of CXPA presenting positivity for tenascin showed metastases at some point during the follow-up period (70) (Table 1). Furthermore, tenascin seems to be important for CXPA tumor invasion and progression. In the invasive areas, tenascin showed strong expression on the tumor front of intracapsular and minimally invasive CXPA, while its expression was focal within the tumor. Interestingly, expression was low or negative in frankly invasive CXPA (70).

### Collagen

Collagen represents about 30% of the total proteins in humans and is the most abundant fibrous protein in the interstitial ECMs (16, 23). The collagen superfamily is composed of twenty-eight different types of collagens that differ in structure and properties (71). In cancer, collagen is modified to provide modulation of the TME favoring malignant cellular proliferation (72). Several studies have shown that collagen is an important prognostic factor correlated with cancer invasion, lymph node metastasis, clinical stage, and treatment resistance of various types of cancers, such as esophageal carcinoma, pancreatic carcinoma, colorectal carcinoma, and ovarian carcinoma (73).

In the very first report on the diversity of collagen expression in PA, the authors showed evidence of the relationship between collagen and PA cell proliferation (74) (Table 1). Other studies, however, showed that this tumor presents a heterogeneous pattern of collagen expression. Type I and II collagens compose extracellular collagen crystalloids, structures that can be found in the tumor parenchyma (75). Type II collagen is also present in the PA chondroid areas (49, 76) while type IV collagen is more common in PA with hyaline, fibrous, and chondroid stroma (37). Type VII collagen seems to have a more heterogeneous and diffuse expression in different PA ECMs types (77). When malignant transformation occurs, the interstitial matrix becomes increasingly desmoplastic to allow invasion of cancer cells. Tumor desmoplasia is a feature associated with increased activity of cancer-associated fibroblasts (CAFs) (78). The role of CAFs in CXPA seems to have been explored only recently. A single study available in the literature using immunolabeling of vimentin and α-SMA showed that CAFs are abundantly present in carcinomatous areas of CXPA (79). Several clinical trials targeting CAFs are currently active in different tumors, such as head and neck cancer, gastrointestinal carcinoma, hepatocellular carcinoma, breast carcinoma, melanoma, and other solid tumors (80). This highlights the need for further exploration of the role of CAFs in CXPA and other salivary carcinomas.

What is known so far, is that of all existing collagen types, type I and IV collagens seem to be the most important in the PA malignant transformation. Araújo and colleagues (2009), analyzed epithelial components of CXPA at different stages of invasion. The frankly invasive CXPA showed type I collagen expression among the small nests of tumor cells that embraced the invasive front. In these areas, direct contact of tumor cells with type I collagen could be associated with reduced expression of adhesion molecules E-cadherin and β-catenin and with invasive behavior of ductal epithelial cells (81). On the other hand, in the earliest stages, as in intraductal carcinomas, type I collagen present an amorph and disorganized morphology (81). In this initial stage, type I collagen would act by impairing the function of myoepithelial cells as tumor suppressors, by inducing the increase of fibroblast growth factor 2 (FGF-2.) Indeed, type I collagen has the ability to affect tumor cell behavior (78). In mammary gland cancer, for example, type I collagen is deregulated and is implicated with more invasive behavior and metastasis of tumor cells (82, 83). Type IV collagen appears to be even more important and has already been related to CXPA metastatic behavior, being significantly more expressed in CXPA than in developed metastases (84). Indeed, type IV collagen is involved in tumor invasion and metastasis in other similar models of tumorigenesis, such as colorectal cancer (85) and breast cancer (86) (Table 1).

### Adhesion proteins

#### Integrin

Integrins are transmembrane heterodimers that bind to ECM components providing support for cell motility and invasion (87). There are twenty-four different integrin dimers, each with different tissue and matrix binding specificity (88, 89). Due to their complex action on cellular mechanisms, an alteration in integrin-mediated adhesion and signaling may participate in cancer initiation and progression (87, 90). Crosstalk between integrin and ECM is crucial for maintaining tumor, metastasis, and drug resistance (91). Studies also pointed to a relationship between the activation of these proteins and the maintenance of tumor stem cells (90).

In the normal salivary gland, the expression of integrins and their subunits usually occurs in myoepithelial cells, basal cells, and ductal cells (92, 93). Similar characteristics were found in PA with basically all tumor cells positive for VLA-integrin. Interestingly, this similarity in pattern could suggest a more important pathogenic action of ductal basal cells (92). In a similar fashion to the normal salivary gland, the expression of integrin and all its subunits in PA can be noted in luminal cells and intercellular contacts of myoepithelial cells (94). The role of this protein in PA-CXPA progression has not yet been explored. An increased expression of integrin in malignant salivary tumors, in addition to its relationship with invasion capacity, has already been reported (62). However, more aggressive salivary gland carcinomas (SGCs) seem to present a lower expression of the protein (94) probably due to its deterioration in face of cell adhesion loss (95) (Table 1).

During the evolution of carcinoma *in situ* to an invasive CXPA, the malignant luminal cells normally surrounded by benign myoepithelial cells invade the stromal area, while myoepithelial cells undergo a process of differentiation becoming autophagic and senescent (96). The myoepithelial cell in the autophagy/senescence process may produce metabolites that would be used as energy by the epithelial cells. The epithelial cells may break the basement membrane and invade the adjacent tissues (96, 11).

Recent studies have shown that the loss of integrin-mediated cell adhesion induces autophagy, and it may contribute to the autophagy of myoepithelial cells. As the role of integrin in the PA-CXPA sequence is still unclear, more studies correlating these hypotheses are needed. On the other hand, clinical trials have failed to investigate the efficacy of therapies targeting integrins in prostate cancer, colorectal cancer, melanoma, glioma, and other solid tumors (97). Several promising possibilities of integrins as therapeutic targets await clinical trials, this way studies focused on the heterogeneous expression of integrin in PA-CXPA should be encouraged.

#### Laminin

Laminins are extracellular glycoprotein constituents of the basement membrane responsible for the stratification of the epithelial cells and connective tissue (98, 99). About 16 laminin trimers are reported *in vivo*. The distribution of laminin isoforms depends on tissue type, and they are important for embryogenesis, organogenesis, angiogenesis, and tissue repair (100–102). In tumorigenesis, once activated, these proteins promote cell proliferation, migration, differentiation, and metastasis (98, 99). Indeed, laminin facilitates cell migration and invasion in invasive ductal breast cancer (103), gastric cancer (104), and bladder cancer (105). In addition, laminin may be correlated with the immune response in ovarian cancer (104).

In the normal salivary gland, laminin is expressed in the basement membrane, around the acini and ducts, associated with the presence of the myoepithelial cells (93). In SGCs, laminin expression is reduced, and its discontinuation is frequently observed due to the destruction of the basement membrane in the face of malignancy (93). Similarly, to the normal salivary gland and other SGCs, in PA and CXPA laminin is expressed in the ECM component (67). Studies demonstrated a pattern of intense immunohistochemical labeling around neoplastic cell clusters (37, 84, 106). A similar pattern has also been noted in other SGCs such as adenoid cystic carcinoma and SGCs (67, 106). While CXPA laminin expression is prominent in benign myoepithelial cells surrounding malignant areas, PA laminin expression in myoepithelial cells was shown to be reduced (93, 107). This pattern suggests a change in the myoepithelial cell phenotype during CXPA development (109). With regards to the expression of laminin in PA matrices, this protein is more frequently observed in hyaline matrices when compared to myxoid and chondroid matrices (37, 106). When malignant transformation occurs, laminin expression was shown to be present in both benign and malignant areas of the CXPA (84) (Table 1).

#### Fibronectin

Fibronectins are composed of two subunits covalently connected with disulfide bonds at their C-termini (108). Several stimuli trigger the production of fibronectin matrix fibers, and continuous production is required to maintain the presence of the prior fibronectin matrix (108, 109). In addition to being involved in the stages of morphogenesis, remodeling, and repair (109), the fibronectin matrix plays a favorable role in tumor progression and is dramatically upregulated around the tumor vascular network (16, 23). In head and neck cancer, fibronectin may promote proliferation, migration, and invasion of tumor cells and induces macrophage M2 polarization *in vitro* (110). In breast cancer, fibronectin expression in tumor cells promotes metastasis (111). Pharmacological inhibition of fibronectin in breast cancer slowed cancer progression *in vitro* and *in vivo*, and this highlights its role as adjuvant therapy in these tumors (112).

In PA, fibronectin exhibits strong reactivity in the fibrous stroma (65), especially in the chondromyxoid matrix (66, 67). In PA malignant transformation, in areas of *in situ* carcinoma, fibronectin expression is shown to be increased when compared to areas of residual PA. At this stage, fibronectin may assist in inhibiting myoepithelial cell suppressor function (70) and, therefore, contribute to tumor growth. Fibronectin showed a different expression pattern in different types of CXPA invasion. While in intracapsular CXPA the pattern of the fibronectin expression was found to be around ductal structures, in minimally invasive and frankly invasive tumors the fibers positive for fibronectin showed to be present among the malignant cell nests. This may point to a dynamic performance of the ECM in the progression of the CXPA (89). However, some areas of the tumor edge of intracapsular and minimally invasive CXPA do not express fibronectin. This may highlight the conflicting roles of fibronectin throughout tumor progression (113) (Table 1) and further studies are encouraged to define its role in disease progression, especially given its recent role in cancer therapy (114).

#### Cadherin

The cadherin superfamily is composed of over 100 members there are expressed in the highest levels in distinct tissue types during development (115). In malignant tumors, the cadherins family is downregulated causing a reduction in cell-cell adhesiveness (116). In breast cancer, tumor types of analogs to SGCs, E-cadherin has been associated with invasion and metastasis (117–119).

In the normal salivary gland, cadherins were found in the membrane around acinar and ductal cells, but with absent expression in myoepithelial cells (120, 121). In the case of PA and CXPA, the pattern of expression is similar, with loss of expression in carcinomatous regions associated with loss of cell cohesion and invasion (81, 120, 121). In CXPA methylation of the E-cadherin promoter was related to luminal differentiation, high tumor grade, tumor size, and high TNM stage (122). In contrast, in PA, overexpression of the cadherin-11 subunit has been related to tumor recurrence (123). E-cadherin has already been indicated as a mediator of adenoma-carcinoma progression in pancreatic tumors (124). Few studies have evaluated the relationship of this protein with the PA malignant transformation. Araújo et al. (2009) showed that type I collagen can reduce the membrane expression of E-cadherin in frankly invasive CXPA, and maybe a factor that contributes to tumor invasion (81). Genelhu and collaborators (2007) related the expression of β-catenin—participant of the adhesion complex E-cadherin/catenins—with molecular events in the phenotype transition and initiation of PA-CXPA progression (125) (Table 1).

### MMPs and TIMPs

MMPs are a group of zinc-dependent endopeptidases related to the degradation and remodeling of the ECM. To date, 23 MMPs have been identified and many are implicated in cancer, especially MMP-1, MMP-2, MMP-3, and MMP-9 (126, 127). MMPs can degrade the protein components of the ECM and basement membrane, facilitating tumor invasion and progression (126, 128). MMP-2 and MMP-9 are perhaps the most studied. MMP-2 is known to cleave tenascin. MMP-9 can degrade laminin, collagen IV, and FN (129). The action of MMPs is regulated by the interaction of tissue inhibitors: TIMP-1, TIMP-2, TIMP-3, and TIMP-4 (130). Alterations in TIMPs are present in all human cancers and play an important role not only in the TME but also in cellular interaction with cytokines and growth factors (130, 131).

A recent meta-analysis evaluated the relationships of variants in MMP-2, MMP-7, and MMP-9 for cancer risk (132). The results of this work showed that MMP-2 rs243865 is most correlated with esophageal cancer and lung cancer, MMP-7 rs11568818 with bladder and cervical cancer, and MMP-9 rs3918242 with breast cancer. In breast cancer, the pattern of expression of MMPs can assist in tumor prognostic classification. It has been reported that neoplasms with high expression of TIMPs were correlated with a more indolent clinical course and good prognosis, while tumors with high expression of MMPs were correlated with a more aggressive clinical course and increased risk of recurrences (131).

In SGTs, the expression of MMPs have been shown similarities across benign tumors (133) while in carcinomas, the imbalance between MMPs/TIMPs is associated with tumor invasion and metastasis. Overexpression of MMP-2 was associated with more aggressive behavior (134), while MMP-7, MMP-9, and MMP-13 are related to a poor prognosis (135–137). The positive expression of the MMPs inducer (EMMPRIN) was considered an angiogenic factor in these tumors, reinforcing the promoting role of MMPs in the induction, maintenance, and progression of SGTs (138).

Regarding PA, MMP-2, MMP-9, TIMP-1, and TIMP-2 seem to control local invasiveness *in vitro* (139). A study using ELISA compared MMPs and TIMPs in the salivary fluid of patients with PA and salivary SGCs. Their results showed that MMP-8, TIMP-1, and TIMP-2 are important biomarkers for the diagnosis of PA (140). Although there is a high expression of MMP-2 and MMP-9 in the myoepithelial cells of the PA, the expression of MMP-2 is significantly higher and more stable than MMP-9 (141). Regarding MMP-2, *in vitro* assays showed that its action leads to the degradation of the basement membrane and contact of myoepithelial cells with the tumoral stroma. This mechanism may be dependent on EGF interaction with tumor cells that act to modify the expression of the E-cadherin/β-catenin complex (142, 143).

Given the MMPs changes found in PA and cancers, we could hypothesize that MMPs may play a promoting role in the PA malignant transformation, with the myoepithelial cells as a key component (70). Corroborating with this hypothesis, a recent study by Martinez et al. showed an increase in *MMP-2* and *MMP-9* and decreased *TIMP-2* mRNA and protein expression when myoepithelial cells were exposed to epithelial cells exosomes *in vitro* (144) (Table 1).

With regards to TIMPs in PA and other SGCs, different studies have shown that the increase in MMPs expression is followed by an increase in TIMP expression (especially TIMP-1 and TIMP-2) and this may represent a regulatory mechanism to maintain the balance of the MMPs/TIMPs ratio (141, 145).

## Conclusions and future directions

In summary, in this work, we reviewed the complexity of the ECM in the CXPA development and progression. The studies summarized here have revealed how the heterogeneity and versatility of the ECM in the PA and during its malignant transformation may affect the biology and behavior of these tumors. Important changes in the components of the ECM in this class of tumors including the presence of perlecan in PA and its association with tumor recurrence as well as the higher expression of fibronectin, collagen type I, and IV, and lower expression of e-cadherin in the CXPA development. As in other cancer types, this review reinforced the perceptions and addressed PA-CXPA not only as a disease of cell transformation and uncontrolled proliferation but also as changes in TME that undergo dynamic remodeling during all stages of the PA-CXPA sequence. Further knowledge of the role of ECM in this entity may provide tools for promising therapeutic targets hampering cancer cells' ability to metastasize and effectively limit the spread of CXPA malignant cells.
